# Rough set theory based prognostic classification models for hospice referral

**DOI:** 10.1186/s12911-015-0216-9

**Published:** 2015-11-25

**Authors:** Eleazar Gil-Herrera, Garrick Aden-Buie, Ali Yalcin, Athanasios Tsalatsanis, Laura E. Barnes, Benjamin Djulbegovic

**Affiliations:** 1 Threshold Tuning Optimization Team Citigroup, 3800 Citibank Center, Tampa, 33610 FL USA; 2 Department of Industrial and Management Systems Engineering, University of South Florida, 4202 E. Fowler Avenue, ENB 118, Tampa, 33620 FL USA; 3 Department of Systems and Information Engineering, University of Virginia, 151 Engineer’s Way, Charlottesville, 22904 VA USA; 4 Department of Hematology and Health Outcomes and Behavior, H. Lee Moffitt Cancer Center & Research Institute, University of South Florida, 12902 Magnolia Drive, Tampa, 33612 FL USA

**Keywords:** Rough set theory, Dominance-based rough set approach, Hospice referral, Prognostic models

## Abstract

**Background:**

This paper explores and evaluates the application of classical and dominance-based rough set theory (RST) for the development of data-driven prognostic classification models for hospice referral. In this work, rough set based models are compared with other data-driven methods with respect to two factors related to clinical credibility: accuracy and accessibility. Accessibility refers to the ability of the model to provide traceable, interpretable results and use data that is relevant and simple to collect.

**Methods:**

We utilize retrospective data from 9,103 terminally ill patients to demonstrate the design and implementation RST- based models to identify potential hospice candidates. The classical rough set approach (CRSA) provides methods for knowledge acquisition, founded on the relational indiscernibility of objects in a decision table, to describe required conditions for membership in a concept class. On the other hand, the dominance-based rough set approach (DRSA) analyzes information based on the monotonic relationships between condition attributes values and their assignment to the decision class. CRSA decision rules for six-month patient survival classification were induced using the MODLEM algorithm. Dominance-based decision rules were extracted using the VC-DomLEM rule induction algorithm.

**Results:**

The RST-based classifiers are compared with other predictive and rule based decision modeling techniques, namely logistic regression, support vector machines, random forests and C4.5. The RST-based classifiers demonstrate average AUC of 69.74 % with MODLEM and 71.73 % with VC-DomLEM, while the compared methods achieve average AUC of 74.21 % for logistic regression, 73.52 % for support vector machines, 74.59 % for random forests, and 70.88 % for C4.5.

**Conclusions:**

This paper contributes to the growing body of research in RST-based prognostic models. RST and its extensions posses features that enhance the accessibility of clinical decision support models. While the non-rule-based methods—logistic regression, support vector machines and random forests—were found to achieve higher AUC, the performance differential may be outweighed by the benefits of the rule-based methods, particularly in the case of VC-DomLEM. Developing prognostic models for hospice referrals is a challenging problem resulting in substandard performance for all of the evaluated classification methods.

## Background

Hospice care reduces the emotional burden of illness on terminal patients by optimizing pain relief strategies [1] and provides a demonstrated, cost-effective increase in the quality of end-of-life care when compared to conventional programs [2]. This increase in quality of care elevates the quality of life of both patients and their families [3].

The advantages of hospice care are diminished for terminally ill patients who enter either prematurely or too late. In general, premature hospice referral represents a lost opportunity for the patient to receive potentially effective and life-prolonging treatment. Conversely, late hospice referral is not desirable and negatively impacts both the quality of end-of-life care and the quality of life of patients and their families [4, 5]. According to Medicare regulations, patient eligibility for hospice care is contingent upon a life expectancy of less than six months, as estimated by the attending physician and certified by the medical director of the hospice program [6]. Medicare claims data report that 14.9 % of hospice care patients lived for more than 180 days after enrollment, while 28.5 % were late referrals who died within 14 days [4, 6]. Accurate prognostication of life expectancy is crucial in end-of-life care decisions and is consequently of vital importance for patients, their physicians and their families.

Prognostic models are an important instrument in prognostication as, in conjunction with direct physician observation, they increase the accuracy of prognostication when compared to physician observation alone [7]. However, a significant barrier to the widespread practical use of prognostic models is their perceived lack of clinical credibility [8].

The objective of this work is to explore and evaluate the application of rough set approaches in the development of data-driven prognostic models with respect to two criteria essential to clinical credibility: accuracy and accessibility. To this end, we will explore Rough Set Theory (RST) as it is applied to end-of-life care and hospice referral decision support models. Additionally, we will compare the results of the RST-based models with several widely known methods: logistic regression, support vector machines (SVM), C4.5, and random forests (RF).

This paper is organized as follows: The Motivation Section presents important features of clinically credible prognostic models and other characteristics of clinical data sets that motivate the use of RST. We then present an overview of the fundamental theory of rough sets for analyzing datasets (section Methods), followed with a similar overview of the theory of the Dominance-based Rough Set Approach (DRSA). In this section, we also discuss the use of decision rules in conjunction with the rough set approaches. The section Dataset description describes the dataset used for the demonstration of the proposed prognostic models. Section Experimental design presents the development of the prognostic models, followed by an overview of the performance evaluation methods used in this study. Finally, we report the results and conclusions, and discuss limitations and future directions of our work.

## Motivation

The objective of a prognostic model is to determine relationships between covariates and a health-related outcome. In the case of life expectancy estimation, prognostic models improve the accuracy in critical clinical decisions and are shown to be superior to physicians’ prognostication alone [9]. Models for estimating the life expectancy of terminally ill patients include the use of statistical and probabilistic methods [10–18], artificial intelligence techniques such as neural networks and support vector machines (SVM) [19–21], decision trees [22, 23] and rough set methods [24, 25]. Survival models [6, 12, 14, 16, 18, 22, 23] focus on estimating the probability that a patient will survive a finite period of time. Classification models, based on methods such as neural networks, SVM and logistic regression [17, 19–21, 26], represent the survival outcome as a binary variable, predicting the status of a patient at a critical point in time (e.g. six months) by classifying the patient as surviving or not surviving the critical time frame. Classification models require the use of non-censored data where survival outcome is known for every patient in the dataset at the critical decision point in time.

A recent review [15] demonstrated that, despite the importance of accurate prognostication within the spectrum of medical care objectives, there is a lack of accessible and accurate prognostic models available to physicians in practice. To withstand clinical trials, and to meet the needs of physicians and patients, a prognostic model must have clinical credibility, meaning that the model must posses a high level of accuracy and accessibility for physicians to believe in the value of the model as a prognostic tool. That is, in addition to accurate prognostication, such a model should be traceable in its structure, meaning the “model’s structure should be apparent and its predictions should make sense to the doctors who will rely on them” [8]. Likewise, the model should provide interpretable results that facilitate explanation of the prognosis, the data required for the model must be relevant and simple to collect with high reliability, and physicians must be able to apply the modeling method correctly without violating the fundamental assumptions of the model.

Clinical datasets present unique challenges that must also be addressed when building data-driven prognostic models. Cios and Moore [27] argue that there are a number of features specific to medical data that result from the volume, heterogeneity and complexity of data that lack canonical form. Additionally, ethical, legal and societal concerns greatly affect the framework under which medical data may be used. The current US model encourages the use of de-identified, minimal risk medical data for research purposes, specifically data collected during routine treatment of patients. It is common for medical data collected in such a way to contain redundant, insignificant, incomplete or inconsistent data objects. Furthermore, the underlying conceptual structures of medicine are not easily formalized mathematically, as the medical field lacks the necessary constraints for the mathematical characterizations common to the physical sciences. As a result, many medical concepts are vaguely defined [28].

Rough Set Theory [29] is a mathematical tool for data analysis that has been used to address vagueness and inconsistencies present in datasets [30]. RST provides a systematic approach for analyzing data without implicit assumptions about relationships between covariates, an advantage that makes RST suitable for integration into medical applications [31]. The information extracted from the dataset by RST and its related methods can be represented in the form of “if–then” decision rules—an intuitive representation that offers significant advantage over “black box” modeling approaches [32] and that increases accessibility and thus clinical credibility.

In the medical field, applications of RST focus mainly on the diagnosis and prognostication of diseases, where it has been demonstrated that RST is useful for extracting medical prognostic rules from minimal information. Tsumoto [33] argues that the concepts of approximation established in RST reflect the characteristics of medical reasoning, explaining why RST performs well in the medical field. For example, RST can be used to highlight non-essential prognostic factors in a particular diagnosis, thus helping to avoid redundant, superfluous or costly tests [34–38]. Recently, methods that combine survival analysis techniques and RST have been used to generate prognostic rules that estimate the survival time of a patient [24, 25].

## Methods

### Classical rough set approach (CRSA)

Rough Set Theory, introduced by Pawlak in [29], provides methods for generalizing or reducing information so as to facilitate knowledge discovery by exploiting the relational indiscernibility of objects in an information table. Central to RST is the notion that an observed object has a certain amount of information associated with it. When considered in relation to a cohort of observed objects, this information is used to group similar objects into information granules. Together, the information provided by the set of observed objects can be generalized to describe the conditions required for membership in a concept class.

#### Notation

The methods of classical RST, hereafter referred to as the CRSA, act upon an information table of the form *S*=(*U,A,V,f*), where *U* is a non-empty finite set of objects, called the universe. *A*=*C*∪{*d*} is a set of attributes that describe a given object in *U*, comprised of a set *C* of condition attributes and an optional decision attribute *d*. When *d* is present, the information table is a decision table. The set of all values, *V*, contains the value sets *V*_*a*_, for every attribute *a*∈*A*. Given an object *x*∈*U*, *f*:*U*×*A*→*V* maps the condition attribute of object *x* to its associated value *v*=*f*(*x,a*)∈*V*_*a*_. A value attribute pair (*a,v*) for a given object is referred to as a descriptor.

Table 1 provides an example of a discretized decision table, where six prognostic factors, as the condition attributes, describe seven patients. The decision attribute, presence of coronary disease in the patient, is represented by the binary attribute *d*→{Yes,*No*}.

**Table 1:** Example decision table

	Condition attribute^a^						Decision attribute
	*c*_1_	*c*_2_	*c*_3_	*c*_4_	*c*_5_	*c*_6_	*d*
Patient	Gender	Age	SystBP	HDL	Diabetic	Smoker	Coronary disease
*x*_1_	F	H	M	L	No	No	No
*x*_2_	M	L	L	L	No	Yes	No
*x*_3_	F	M	M	H	No	No	No
*x*_4_	F	M	M	H	No	No	Yes
*x*_5_	M	H	H	L	Yes	Yes	Yes
*x*_6_	M	H	H	L	Yes	Yes	Yes
*x*_7_	F	M	M	H	No	No	Yes

^a^Gender: Female/Male; Age: L = [54,59), M = [59,69), H = [69,74]; SystBP: L =<129, M = [ 129−139], H =(139−159]; HDL: L = <40 M = [ 40−60], H =>60

The objects in a decision table can be grouped according to their descriptors. For example, patients *x*_5_ and *x*_6_ have the same attribute values and are thus indiscernible from each other. In general, two objects *x*_*i*_,*x*_*j*_∈*U* are indiscernible with respect to a set of condition attributes *B*⊆*C* if *f*(*x*_*i*_,*a*)=*f*(*x*_*j*_,*a*) ∀*a*∈*B*. This relation is called an indiscernibility relation, defined as *R*(*B*)={(*x*_*i*_,*x*_*j*_)∈*U*:∀*a*∈*B,f*(*x*_*i*_,*a*)=*f*(*x*_*j*_,*a*)}.

For example, the patients in Table 1 can be separated into four groups according to the indiscernibility relation *R*(*C*):*X*_1_={*x*_1_},*X*_2_={*x*_2_},*X*_3_={*x*_3_,*x*_4_,*x*_7_},*X*_4_={*x*_5_,*x*_6_}. These groups of objects are referred to as equivalence classes, or conditional classes for *B*⊆*C*. An equivalence class for the decision attribute is called a decision class or concept, and in this example there are two groups: *Y*_*No*_={*x*_1_,*x*_2_,*x*_3_} and *Y*_*Yes*_={*x*_4_,*x*_5_,*x*_6_,*x*_7_}. The equivalence class specified by the object *x*_*i*_ with respect to *R*(*B*) is denoted as [ *x*_*i*_]_*B*_.

#### Set approximations

The goal of the CRSA is to provide a definition of a concept according to the values of the attributes of the equivalence classes that contain objects that are known instantiations of the concept. As such, in a consistent decision table, membership in a conditional class implies membership in a particular decision class. In Table 1, *x*∈*X*_4_ implies *x*∈*Y*_*Yes*_. Membership in *X*_3_, however, does not imply *Y*_*Yes*_ as *x*_4_,*x*_7_∈*Y*_*Yes*_ but *x*_3_∈*Y*_*No*_. Thus Table 1 is inconsistent as *f*(*x*_4_,*d*)≠*f*(*x*_3_,*d*) and *f*(*x*_7_,*d*)≠*f*(*x*_3_,*d*).

To represent an inconsistent decision table, the CRSA establishes an upper and lower approximation for each decision class, *Y*. The lower approximation is comprised of all objects that definitely belong to *Y*, while the upper approximation includes all objects that possibly belong to *Y*. It can be said that an object *x*_*i*_ definitely belongs to a concept *Y* if [ *x*_*i*_]_*C*_⊆*Y* and that *x*_*i*_ possibly belongs to a concept *Y* if [ *x*_*i*_]_*C*_∩*Y*≠*∅*. Thus, the lower and upper approximations are defined as follows:

$$\begin{array}{@{}rcl@{}} \underline{R}_{B} (Y) & =& \left\lbrace x \in U \colon [\!x]_{B} \subseteq Y \right\rbrace = \bigcup \left\lbrace [\!x]_{B} \colon [\!x]_{B} \subseteq Y \right\rbrace \\ \overline{R}_{B} (Y) &=& \left\lbrace x \in U \colon [\!x]_{B} \cap Y \neq \emptyset \right\rbrace = \bigcup \left\lbrace [\!x]_{B} \!\colon\! [\!x]_{B} \cap Y \neq \emptyset \right\rbrace  \\ \overline{R}_{B} (Y) &- &\underline{R}_{B} (Y) = BND_{B} (Y)  \end{array} $$

The boundary region, *BND*_*B*_(*Y*), contains those objects that possibly, but not certainly, belong to *Y*. Conversely, the set $U - \overline {R}_{B} (Y)$ is the outside region containing those objects that certainly do not belong to *Y*. In our example, the lower and upper approximations for *Y*_*Yes*_ are $\underline {R}_{C} (Y_{\mathit {Yes}}) = X_{4} = \{ x_{5}, x_{6} \}$ and $\overline {R}_{C} (Y_{\mathit {Yes}}) = X_{4} \cup X_{3} = \{ x_{3}, x_{4}, x_{5}, x_{6}, x_{7} \}$, and the boundary region contains the objects *BND*_*B*_(*Y*_*Yes*_)={*x*_3_,*x*_4_,*x*_7_}.

Let *F*={*Y*_1_,*Y*_2_,…,*Y*_*n*_} represent a classification, i.e. a set of decision classes. The quality of approximation of classification, *γ*_*B*_(*F*), with respect to attributes *B*, expresses the ratio of all objects covered by the lower approximation *R̲*_*B*_(*F*) = {*R̲*_*B*_(*Y*_1_),*R̲*_*B*_(*Y*_2_),…,*R̲*_*B*_(*Y*_*n*_)} over all objects in *U*. The quality of approximation is expressed as:

$$\gamma_{B} (F) = \frac{\sum_{t=1}^{n} \left| \underbar{R}_{B} (Y_{t}) \right|}{\left| U \right|} $$

### Dominance-based rough set approach (DRSA)

Under the DRSA [39] the relations between objects are no longer made by the indiscernibility relation as described in the CRSA [29]. In its place, the DRSA introduces a new dominance relation that allows for ordinal attributes with preference-ordered domains wherein a monotonic relationship exists between the attribute and the decision classes. An example of such a relationship occurs when a “better” or “worse” value of an attribute leads to a “better” or “worse” decision class.

#### Notation

A decision table in the DRSA is expressed in the same way as the CRSA. To differentiate between attributes with and without a preference-ordered domain, those with a preference order are called criteria while those without are referred to as attributes, as in the CRSA.

In the DRSA the domain of criteria *a*∈*A* is completely preordered by the outranking relation ≽_*a*_, representing the preference order of the domain. The outranking relation is also applicable for comparing two objects such that for *x*_*i*_,*x*_*j*_∈*U*, *x*_*i*_≽_*a*_*x*_*j*_ means that *x*_*i*_ is at least as good as (outranks) *x*_*j*_ with respect to the criterion *a*∈*A*.

Commonly, the domain of a criteria *a* is a subset of real numbers, *V*_*a*_⊆*R* and the outranking relation is then a simple order “ ≥” on real numbers such that the following relation holds: *x*_*i*_≽_*a*_*x*_*j*_⇔*f*(*x*_*i*_,*a*)≥*f*(*x*_*j*_,*a*). This relation is straightforward for gain-type criteria (the more, the better), and can be easily reversed for cost-type criteria (the less, the better).

Using Table 1 as an example, the decision criterion *d* is preference-ordered such that a positive diagnosis of coronary disease is assumed to be the “preferred” decision class. Criterion preference relations are then organized in the direction of the decision class; values which generally contribute to the incidence of coronary disease are preferred over those which indicate lower risk, much in the same way that a positive diagnosis indicates presence of coronary disease. For the criteria in Table 1, higher values are preferred to lower values—as in the case of *Age*, *SystBP*, and *HDL*—and “Yes” is preferred to “No”—as in the case of *Smoker* and *Diabetic*. No such preference relation exists for *Gender*; as such, it is considered an attribute.

Let *T*={1,…,*n*} represent increasing indexes corresponding to the order of preferences of the decision criterion *d*. Then, the decision table is partitioned into *n* classes *Y*_*t*_, *t*∈*T*, where each object *x*∈*U* is assigned to one and only one class *Y*_*t*_. The decision classes are preference-ordered according to the decision maker, i.e. for all *r,s*∈*T* such that for *r*>*s* the objects from class *Y*_*r*_ are strictly preferred to the objects from class *Y*_*s*_.

For our example in Table 1, *Y*_1_={*x*_1_,*x*_2_,*x*_3_} corresponds to patients without a coronary disease and *Y*_2_={*x*_4_,*x*_5_,*x*_6_,*x*_7_} corresponds to the patients with a coronary disease. Therefore, each patient in *Y*_2_ is preferred to each patient in *Y*_1_.

#### Set approximations

In the DRSA, the approximated sets are upwards and downwards unions of decision classes rather than individual decision classes as in the CRSA. Upward and downward unions of classes are defined as:

$$ Y_{t}^{\geq} = \bigcup_{s \geq t} Y_{s} \text{\quad and \quad} Y_{t}^{\leq} = \bigcup_{s \leq t} Y_{s}, \; s,t \in T $$

For any pair of objects (*x*_*i*_,*x*_*j*_)∈*U*, *x*_*i*_ dominates *x*_*j*_ with respect to a set of condition attributes *P*⊆*C*, denoted by *x*_*i*_ *D*_*P*_ *x*_*j*_, if the following conditions are satisfied simultaneously:

$$\begin{array}{@{}rcl@{}} & x_{i} \succeq_{q} x_{j}, &\text{for all critera}~~q \in P  \\ f(x_{i},a) & = f(x_{j},a), &\text{for all attributes}~~a \in P  \end{array} $$

The dominance relation defines two sets called dominance cones, where for each *x*_*i*_∈*U*:

$$\begin{array}{@{}rcl@{}} &D_{P}^{+} (x_{i}) = &\{ x_{j} \in U \colon x_{j} \: D_{P} \: x_{i} \}, \: \text{representing the set of}\\&& \text{objects that dominates}~~ x_{i}  \\ &D_{P}^{-} (x_{i}) = & \{ x_{j} \in U \colon x_{i} \: D_{P} \: x_{j} \}, \: \text{representing the set of}\\&& \text{objects dominated by}~~ x_{i}  \end{array} $$

Considering the dominance cones, the lower and upper approximations of the union of decision classes are defined as follows. The lower approximation $\underline {R}_{P} \left (Y_{t}^{\geq }\right)$ represents objects that certainly belong to $Y_{t}^{\geq }$, such that there is no other object that dominates *x* and belongs to a decision class inferior to *Y*_*t*_. Similarly, the lower approximation $\underline {R}_{P} \left (Y_{t}^{\leq }\right)$ represents objects that certainly belong to $Y_{t}^{\leq }$, with no other object dominated by *x* and belonging to a decision class superior to *Y*_*t*_. The upper approximations represent objects that possibly belong to one of the upward or downward unions of decision classes.

(1) $$\begin{array}{@{}rcl@{}} \underline{R}_{P} \left(Y_{t}^{\geq} \right) &=&\left\{ x \in U \colon D_{P}^{+} (x) \subseteq Y_{t}^{\geq} \right\} \\ \overline{R}_{P} \left(Y_{t}^{\geq} \right) & = & \bigcup_{x \in Y_{t}^{\geq}} D_{P}^{+}(x) = \left\{x \in U \colon D_{P}^{-} (x) \cap Y_{t}^{\leq} \neq \emptyset \right\} \\ \underline{R}_{P} \left(Y_{t}^{\leq} \right) & = & \left\{ x \in U \colon D_{P}^{-} (x) \subseteq Y_{t}^{\leq} \right\} \\ \overline{R}_{P} \left(Y_{t}^{\leq} \right) & = & \bigcup_{x \in Y_{t}^{\leq}} D_{P}^{-} (x) = \left\{x \in U \colon D_{P}^{+} (x) \cap Y_{t}^{\geq} \neq \emptyset \right\} \\ \end{array} $$

Similar to the CRSA, the boundary regions are defined as:

$$\begin{array}{@{}rcl@{}} BND_{P} Y_{t}^{\geq} = & \overline{R}_{P} \left(Y_{t}^{\geq} \right) - \underline{R}_{P} \left(Y_{t}^{\geq} \right) \\ BND_{P} Y_{t}^{\leq} = & \overline{R}_{P} \left(Y_{t}^{\leq} \right) - \underline{R}_{P} \left(Y_{t}^{\leq} \right)  \end{array} $$

Using our example decision table, Table 1, and considering the full set of condition attributes, it can be seen that *x*_4_ *D*_*C*_ *x*_3_, and furthermore $D_{C}^{+} (x_{4}) = \{ x_{3}, x_{4}, x_{7} \}$, $D_{C}^{-} (x_{4}) = \{ x_{3}, x_{4}, x_{7} \}$. Considering the dominance cones for all patients, the lower and upper approximations of the union of decision classes are $\underline {R}_{C} \left (Y_{2}^{\geq } \right) = \{ x_{5}, x_{6} \}$, $\overline {R}_{C} \left (Y_{2}^{\geq }\right) = \{ x_{3}, x_{4}, x_{5}, x_{6}, x_{7} \}$, $\underline {R}_{C} \left (Y_{1}^{\leq }\right) = \{ x_{1}, x_{2} \}$, $\overline {R}_{C} \left (Y_{1}^{\leq } \right) = \{ x_{1}, x_{2}, x_{3}, x_{4}, x_{7} \}$ and the boundary regions are $BND_{C} Y_{2}^{\geq } = BND_{C} Y_{1}^{\leq } = \{ x_{3}, x_{4}, x_{7} \}$.

For every subset of attributes *P*⊆*C*, the quality of approximation of the decision classes *Y* with respect to the attributes *P*, *γ*_*P*_(*Y*), is defined as the proportion among all objects in *U* of objects consistently defined with respect to the attributes *P* and the decision classes *Y*.

$${} \gamma_{P}(Y) = \frac {\left | U - \left\{\bigcup \limits_{t \in T} BND_{P} Y_{t}^{\leq} \right\} \right |} {|U|} = \frac {\left | U - \left\{\bigcup \limits_{t \in T} BND_{P} Y_{t}^{\geq} \right\} \right |} {|U|} $$

#### The variable consistency DRSA

The variable consistency DRSA (VC-DRSA) allows the decision maker to relax the strictness of the dominance relation, thus accepting a limited number of inconsistent objects in the lower approximation, according to an object consistency level threshold, *l*∈(0,1]. In practice, by selecting this consistency level *l*, a patient *x*∈*U* becomes a member of the lower approximation of a given upward union if at least *l*∗100 *%* of the patients dominating *x* also belong to that decision class. By allowing inconsistencies, the VC-DRSA avoids over fitting the training set and thus may be more effective in classifying new cases.

The lower approximations of the VC-DRSA-based model are represented as follows:

$$\begin{array}{@{}rcl@{}} \underline{R}_{P}^{l} \left(Y_{t}^{\geq} \right) & = & \left\{ x \in Y_{t}^{\geq} \colon \frac{| D_{P}^{+} (x) \cap Y_{t}^{\geq} |}{ | D_{P}^{+} (x) |} \geq l \right\}\\ \underline{R}_{P}^{l} \left(Y_{t}^{\leq} \right) & = & \left\{ x \in Y_{t}^{\leq} \colon \frac{| D_{P}^{-} (x) \cap Y_{t}^{\leq} | }{ | D_{P}^{-} (x) |} \geq l \right\} \\ \end{array} $$

Continuing with the example described in Table 1, setting *l*=0.6 moves the objects *x*_4_ and *x*_7_, previously included in the upper approximation $\overline {R}_{C} \left (Y_{2}^{\geq }\right)$, to the lower approximation of class $Y_{2}^{\geq }$, i.e: $\underline {R}_{C}^{0.6} \left (Y_{2}^{\geq } \right) = \left \{ x_{4}, x_{5}, x_{6}, x_{7} \right \}$. This follows from $\frac {| D_{C}^{+} (x_{i}) \cap Y_{t}^{\geq } |}{ | D_{C}^{+} (x_{i}) |} = \frac {2}{3} \geq l$, for *i*=4,5,6,7.

### Decision rules

There are a number of methods available for induction of decision rules from the lower or upper approximations of the decision classes [40–42] or from reducts extracted from the decision table [43]. Decision rules in this study were obtained using the MODLEM [40, 41] and VC-DomLEM [42] algorithms for the induction of classical and dominance-based rough set rules, respectively. In both cases, decision rules are induced from approximations of decision classes. Both the MODLEM and VC-DomLEM algorithms generate a minimal set of decision rules using a minimal number of rule conditions, thus the inclusion of MODLEM allows for an evaluation of the impact of accounting for the preference order information in the VC-DRSA. Once decision rules have been induced, the collection of these rules can then be used to classify unseen objects—in the case of our example table, a new patient who may have cardiac disease.

A decision rule has the form *if A then B*, or *A*→*B*, where *A* is called the antecedent and *B* the consequent of the rule. The antecedent is a logical conjunction of descriptors and the consequent is the decision class or union of decision classes suggested by the rule.

Formally, in the CRSA, decision rules are generated from the lower or upper approximations. For example, for an approximation containing objects with descriptors *r* with respect to a set of condition attributes, *B*_*r*_⊆*C*, a decision rule is expressed as

$$\mathit{if} \: \bigwedge_{i} \left(\; f(x, a_{i}) = r_{a_{i}} \right) \: \mathit{then} \: x \in Y_{t} $$

where *a*_*i*_∈*B*_*r*_ is an attribute found in the attribute set *B*_*r*_, and $r_{a_{i}} \in V_{a_{i}}$ and *Y*_*t*_ are the attribute values and a decision class, respectively, of the objects in the rule-generating approximation. From our example in Table 1, a decision rule induced with MODLEM from the lower approximation $\underline {R}_{\{Age, Smoker\}} (Y_{\mathit {Yes}}) = \{ x_{5}, x_{6} \}$ would be: if *Age*=H and *Smoker*=Yes then *Coronary* *Disease*=Yes.

In the DRSA, decision rules are induced from the lower approximations and the boundaries of the union of decision classes. From the lower approximations, two types of decision rules are considered. Decision rules generated from the *P*-lower approximation of the upward union of decision classes $Y_{t}^{\geq }$ are described by

$${} {\fontsize{8.9pt}{9.6pt}\selectfont{\begin{aligned} \mathit{if} \: \left(\! \bigwedge_{i} \left(\;f(x, b_{i}) \geq r_{b_{i}} \!\right) \right) \bigwedge \left(\! \bigwedge_{j} \left(\;f(x, a_{j}) = r_{a_{j}} \right)\! \right) \: \mathit{then} \: x \in Y_{t}^{\geq} \end{aligned}}} $$

where *b*_*i*_∈*P* are criteria, *a*_*j*_∈*P* are attributes, $r_{b_{i}} \in V_{b_{i}}$ and $r_{a_{j}} \in V_{a_{j}}$. From the example in Table 1, the *P*-lower approximation of the upward union of the decision class, $Y_{2}^{\geq } = \underline {R_{C}} Y_{2} = \{x_{5}, x_{6}\}$, leads to the following decision rule:

- *If Gender = M and Age ≥ H and HDL ≥ L and Diabetic ≥ Yes and Smoker = Yes, then Coronary Disease = Yes*.

Decision rules generated from the *P*-lower approximation of the downward union of classes $Y_{t}^{\leq }$ are described by

$${} {\fontsize{8.8pt}{9.6pt}\selectfont{\begin{aligned} \mathit{if} \: \left(\bigwedge_{i} \left(f(x, b_{i}) \leq r_{b_{i}} \right) \right) \bigwedge \left(\bigwedge_{j} \left(f(x, a_{j}) = r_{a_{j}} \right) \right) \: \mathit{then} \: x \in Y_{t}^{\leq} \end{aligned}}} $$

where *b*_*i*_∈*P* are criteria, *a*_*j*_∈*P* are attributes, $r_{b_{i}} \in V_{b_{i}}$ and $r_{a_{j}} \in V_{a_{j}}$. From the example in Table 1, the *P*-lower approximation of the downward union of classes $Y_{1}^{\leq } = \underline {R_{C}} Y_{1} = \{x_{1}, x_{2}\}$, leads to the following decision rules:

- *If Gender = F and Age ≤ H and SystBP ≤ M and HDL ≤ L and Diabetic ≤ No and Smoker ≤ Yes, then Coronary Disease = No*
- *If Gender = M and Age ≤ H and SystBP ≤ M and HDL ≤ L and Diabetic ≤ No and Smoker ≤ Yes, then Coronary Disease = No*

The boundaries $BND_{P} Y_{t}^{\geq }$ and $BND_{P} Y_{t}^{\leq }$ generate the following rules

$$\begin{array}{@{}rcl@{}} \mathit{if} \: \left(\bigwedge_{i} \left(\;f(x, b_{i}) \geq r_{b_{i}} \right) \right) \bigwedge \left(\bigwedge_{j} \left(f(x, b_{j}) \leq r_{b_{j}} \right) \right) \\ \bigwedge \left(\bigwedge_{k} \left(\;f(x, a_{k}) = r_{a_{k}} \right) \right) \: \mathit{then} \: x \in Y_{t} \cup Y_{t+1} \cup \ldots \cup Y_{s}  \end{array} $$

where *b*_*i*_,*b*_*j*_∈*P* are criteria, *a*_*k*_∈*P* are attributes, $r_{b_{i}} \in V_{b_{i}}$, $r_{b_{j}} \in V_{b_{j}}$ and $r_{a_{k}} \in V_{a_{k}}$ (note *i* and *j* are not necessarily different). From the example in Table 1, the boundary decision classes $BND Y_{2}^{\geq } = BND Y_{1}^{\leq } = \{x_{3}, x_{4}, x_{7}\}$, leads to the following decision rule:

- *If Age ≥ M and SystBP ≥ M and HDL ≥ H and Diabetic ≥ No and Smoker ≥ No and Age ≤ M and SystBP ≤ M and HDL ≤ H and Diabetic ≤ No and Smoker ≤ No and Gender = F, then Coronary Disease = (No, Yes)*

The MODLEM and the VC-DomLEM algorithms utilize a heuristic strategy called *sequential covering* [44] to iteratively construct a minimal set of minimal decision rules. The sequential covering strategy successively constructs a set of decision rules for each upward and downward union of decision classes in a training set by selecting, at each iteration, the “best” decision rule, after which the training objects described by the rule conditions are removed. Subsequent iterations again select the best decision rule and remove the covered objects until reaching a stopping criteria or until all of the objects in the unions of decision classes are described by a rule in the rule set.

To ensure minimality, antecedent descriptors, called elementary conditions, of each rule are checked at each iteration and redundant elementary conditions are removed. Similarly, redundant rules are removed from the final rule set.

In both algorithms, decision rules are grown by consecutively adding the best available elementary condition to the rule. CRSA elementary conditions are evaluated in the MODLEM algorithm in terms of either the class entropy measure [45] or Laplacian accuracy [46]; the former was used in this study. MODLEM does not restrict elementary conditions to those attributes not currently in the rule; as such, multiple elementary conditions may contain the same attribute. Therefore, a decision rule induced by MODLEM may contain antecedents in which attribute values are described as belonging to a range or a set of values or as being greater or less than a particular value.

Dominance-based elementary conditions are evaluated according to a rule consistency measure. VC-DomLEM provides three such measures; the rule consistency measure used in this study is *μ*, as described in [47]. For the sake of clarity, *Y*_*t*_ shall be used to represent an individual decision class in the CRSA or alternatively an upward or downward union of decision classes, $Y^{\geq }_{t}$ or $Y^{\leq }_{t}$, with respect to the DRSA. The consistency, *μ*, of a proposed rule, $r_{Y_{t}}$, suggesting assignment to *Y*_*t*_ is defined as

$$\mu(r_{Y_{t}}) = \frac{\left| \left[ \Phi (r_{Y_{t}}) \right] \cap \underbar{Y}_{t} \right|} {\left| \left[\Phi(r_{Y_{t}})\right] \right|}. $$

Here $\left [\Phi (r_{Y_{t}})\right ]$ indicates the set of objects described by the elementary conditions in $r_{Y_{t}}$. The elementary condition, *ec*, that is selected for inclusion is that which leads to the highest rule consistency measure $\mu (r_{Y_{t}} \cup ec)$ when combined with the current set of elementary conditions in the proposed rule. In the event of a tie, the elementary condition providing greatest coverage of the new rule is selected, by $\left | \left [ \Phi (r_{Y_{t}} \cup ec) \right ] \cap \underbar {Y}_{t} \right |$. The rule consistency measure, *μ*, was also implemented in MODLEM to relax consistency requirements and to allow more general rules to be induced. For further details on the MODLEM and VC-DomLEM algorithms, the reader is referred to [40–42, 47].

To classify an unseen object, a standard voting process [43] is used to allow all rules to participate in the decision process, arriving at a patient classification by majority vote. Each rule is characterized by two support metrics. The left hand side (LHS) support is the number of patients in the table whose attributes match the antecedent, i.e: |[*Φ*(*r*)]|, while the right hand side (RHS) support indicates the number of patients matching both the antecedent and the consequent of the rule, i.e: |[*Φ*(*r*)]∩*Y*_*t*_|. For a new, unseen patient, any rule whose antecedent descriptors match the patient descriptors “fires” by contributing as votes the RHS support for each decision class. For example, drawing up the example Table 1, the decision rule *If* Age = H *and* Smoker = Yes, *then* Coronary Disease = Yes has LHS = 2 since its antecedent matches patient *x*_5_ and *x*_6_ and RHS = 2 since its antecedent and consequent match the same patients. A new patient matching the antecedent of this rule will receive two votes for decision class *Yes* and zero votes for decision class *No*.

Once all rules have “voted”, the number of votes for each decision class is normalized against the total number of LHS support for all fired rules. The resultant ratio of RHS to LHS support is considered a frequency-based estimate of the probability that the patient belongs to the given decision class.

A final classification is therefore determined according to a threshold value, *τ*∈[ 0,1]. A patient is classified as not surviving six months if the estimated probability of death in six months is greater than *τ*. In the event of an estimated probability equal to *τ*, or in the absence of any fired rules (no rule matches the patient profile), classification is not possible and the patient is labeled *undefined*. For example, if the threshold value is set as 0.5 and the voting process yields an estimated probability of 70 %, then the patient is classified as not surviving the six month period.

### Dataset description

#### SUPPORT dataset

The dataset used in this study is the SUPPORT (Study to Understand Prognoses and Preferences for Outcomes and Risks of Treatments) prognostic model dataset [48], a study of 9,105 terminally ill patients. SUPPORT enrolled patients, 18 years or older, who met specific criteria for one of nine serious illnesses, who survived more than 48 hours but were not discharged within 72 hours. Patients were followed such that survival and functional status were known for 180 days after entry. The result of the SUPPORT study is a prognostic model for 180-day survival estimation of seriously ill hospitalized adults based on cubic splines and a Cox regression model. Given the inclusion criteria (described in full in Appendix 1 of [12]), the dataset is ideal for the present research in regards to clinical applicability, completeness of data, and comparability of results.

We consider as condition attributes the variables used in the SUPPORT prognostic model equation [12] to ensure consistency. The SUPPORT variables include ten physiologic variables in addition to the diagnosis groups, age, number of days in the hospital before entering the study, presence of cancer, and neurologic function as recorded in the SUPPORT data. Attribute names, descriptions and value ranges are listed in Table 2.

**Table 2:** Description of attributes from SUPPORT dataset

Variable name	Description	Patient distribution		
Numerical condition attributes		Range	Mean	Std. Dev.
*age*	Age of the patient	18–101	62.65	15.59
*alb*	Serum albumin	0.4–29	2.95	0.87
*bili*	Bilirubin	0.1–63	2.55	5.32
*crea*	Serum creatinine	0.09–21.5	1.77	1.69
*hday*	Number of days in hospital at study entry	1–148	1.00	9.13
*hrt*	Heart rate	0–300	97.16	31.56
*meanbp*	Mean arterial blood pressure	0–195	84.55	27.70
*pafi*	Blood gasses, *PaO*_2_/(.01∗*FiO*2)	12–890.4	239.50	109.70
*resp*	Respiration rate	0–90	23.33	9.57
*scoma*	SUPPORT coma score, based on Glasgow coma scale	0–100	12.06	24.63
*sod*	Sodium	110–181	137.60	6.03
*temp*	Temperature in °C	31.7–41.7	37.10	1.25
*wblc*	White blood cell count	0.05–200	12.35	9.27
Categorical condition attributes		Patients	Percentage (%)	
*dzgroup*	Diagnosis group:			
	*ARF/MOSF w. sepsis*	3,513	38.59	
	CHF	1,387	15.23	
	Cirrhosis	508	5.56	
	Colon cancer	512	5.62	
	Coma	596	6.54	
	COPD	967	10.60	
	Lung cancer	908	9.97	
	MOSF w. malignancy	712	7.81	
*ca*	Presence of cancer:			
	*Yes*	1,252	13.75	
	*No*	5,993	65.84	
	*Metastasis*	1,858	20.40	
Decision attribute		Patients	Percentage (%)	
*d.6months*	Death occurred within 6 months:			
	*Yes*	4,263	46.83	
	*No*	4,840	53.17	

Values of 0 for *hrt, meanbp* and *resp* correspond to cardiac arrests during the day when the measurements were taken

The median survival time for the patients in the study is 223 days.

**Fig. 1:**
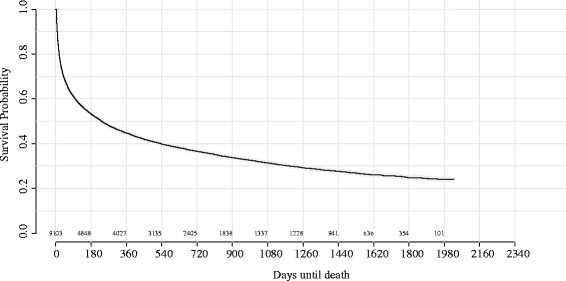
Kaplan-Meier survivability of patients with respect to number of days until death

Figure 1 shows the patients Kaplan-Meier survival curve with respect to number of days until death. The SUPPORT study inclusion criteria was designed to include patients with 50 % risk of death at 180 days; as seen in Table 2 death prior to 180 days was observed in approximately 47 % of patients.

**Fig. 2:**
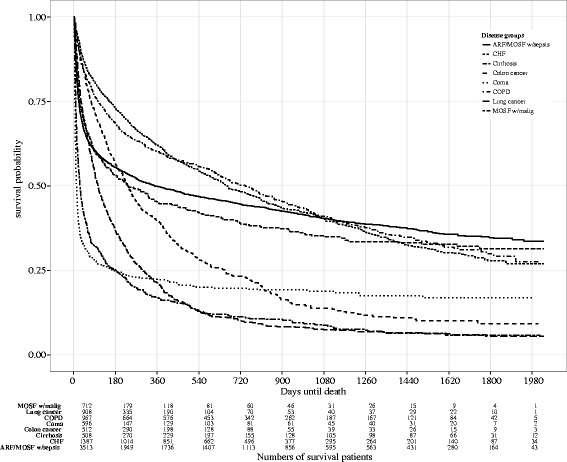
Kaplan-Meier survival curve by *dzgroup*

General observations regarding the influence of condition attributes can be made by analyzing their relation in the proportion of patients surviving the six month period. For example, the Kaplan-Meier survival curve in Fig. 2 shows that a significant portion (75 %) of patients with coma or multi-organ system failure with malignancy (MOSF w/ malig) do not survive longer than 180 days, but patients with congestive heart failure (CHF) or chronic obstructive pulmonary disease (COPD) tend to live longer than 180 days.

#### Data preprocessing

In its published form, the SUPPORT dataset contains 9,105 cases. Missing physiological attribute values are filled in with a standard fill-in value representing a normal physiological response, as provided by the SUPPORT authors in [48]. It is also worth noting that in the SUPPORT study, where neurologic functioning of the patient is recorded in the SUPPORT coma score (*scoma*), a patient for whom it was not possible to establish a Glasgow coma score was given a *scoma* value of zero. After missing data imputation, two cases have missing values in physiological attributes not addressed in the SUPPORT data set. The two incomplete cases were removed and the remaining 9,103 cases were considered in the development of the prognostic models.

#### Discretization

Discretization is the process by which appropriate categorical ranges are found for variables with a continuous value range. There are a number of methods available for unsupervised discretization that operate without input from the decision maker and are based only on the information available in the data table. In this work, however, discretization was primarily performed using the Acute Physiology and Chronic Health Evaluation (APACHE) III scoring system [11], a clinically accepted scoring system designed to estimate the risk of death in ICU patients. In this sense, the use of the APACHE III scoring system represents a research-validated, clinically appropriate, expert discretization scheme. This choice is founded on the proposition that expert discretization via APACHE III will result in medically and contextually relevant classification rules and data collection requirements, thus increasing the accessibility of the proposed prognostic model and ensures directly comparable rule sets for all evaluated rule-based methods.

APACHE III scores are designed to increase monotonically with respect to risk of death and thus provide the necessary preference relations for the DRSA. APACHE III scores for any given variable are close to zero for normal or only slightly abnormal values of that variable and increase according to increased severity of disease. For example, normal pulse rates of 50–99 bpm are given a score of 0, while elevated and lowered levels, 100–109 and 40–49 bpm respectively, are both given a score of 5. Thus, higher APACHE III scores are preferred to lower scores, as the higher scores indicate greater severity of disease and therefore greater risk of death within six months (considered the positive diagnosis). Discretization is not a requirement of any of the methods used in this study, however the APACHE III scores provide the monotonic preference relations for the DRSA and are used for the all of the rule-based methods.

For the rule-based methods considered in this study, the nine physiologic variables and the age variable were transformed to their representative APACHE III scores. The remaining physiologic variables not included in APACHE III—neurologic function, *scoma*, and blood gasses, *pafi*—were discretized using clinically accepted categorizations [49, 50]. The variable *hday* was discretized using the boolean reasoning algorithm [43]. Table 3 shows the categories defined in this process. Higher values of each of these variables are preferred to lower values.

**Table 3:** Discretized attributes not in APACHE III

Attribute	Description	Categorization
*scoma*	Minor	(∗,9]
	Moderate	(9,44]
	Severe	(44,∗)
*pafi*	Normal	[ 300,∗)
	Severe defect in gas exchange	[ 200,300)
	Acute respiratory distress syndrome	[ 0,200)
*hday*	Short	(∗,44]
	Long	(44,∗]

### Experimental design

This section provides details on the implementation and performance evaluation procedures for the comparison of the classification methods used in this study. The following two sections, describe the RSA and comparative methods respectively, the software used for their implementation and the selection of appropriate parameters for each of the methods. Finally, the methods for performance evaluation are discussed.

The general schema of the experimental design is as follows: after selecting appropriate parameters for each of the methods, 5-fold cross validation was used to divide the data into training and testing sets. Methods with decision rule outputs were trained and tested on the discretized data set to demonstrate expected performance of a clinically credible rule set. Methods without decision rule outputs were trained on the raw, non-discretized, data set. For these methods, designed to be applied to continuous variables, discretization does not improve clinical credibility and would likely hinder performance [51, 52].

#### Rough set rule induction and classification

##### MODLEM algorithm for CRSA decision rules

CRSA decision rules were obtained using the MODLEM algorithm as described in [40] and [41], implemented by the authors in the R programming language [53]. Decision rules were generated from the lower approximations with a rule consistency level *μ*≥*m*. The rule syntax follows the presentation in section Decision rules.

##### VC-DomLEM algorithm for VC-DRSA decision rules

Dominance-based rules were obtained using the VC-DRSA as described in section The variable consistency DRSA and the VC-DomLEM algorithm as implemented in jMAF [54]. VC-DomLEM decision rules were generated from the lower approximation of each decision class, with an object consistency level threshold *l*. The syntax of the VC-DRSA decision rules is as shown in section Decision rules. Only decision rules with rule consistency measure *μ* greater than the rule consistency threshold *l* are included in the classification model. Note that the rule consistency threshold and the object consistency threshold are equal and set at *l*.

##### Parameter selection

In order to select the most appropriate models for comparison, the performance of the rough set based models was evaluated for varying levels of rule consistency, *m* and *l*, for the CRSA and VC-DRSA respectively. Classifier performance at a particular value of *m* or *l* is dataset-dependent; however, in general, values close to one provide rule sets that are more conservative in describing the training set objects, while values closer to zero provide rule sets that are more general. Thus, to find the appropriate balance between strict, descriptive models that are prone to overfitting and overly general models that provide little useful information, the RSA models were evaluated at *m,l*=0.1,0.2,0.4,0.6,0.8,1.0.

#### Comparative methods

To evaluate the performance of the RSA-based prognostic models, logistic regression, SVM, and RF were applied to the non-discretized SUPPORT dataset. To ensure directly comparable rule sets, C4.5 was applied to the discretized SUPPORT dataset. Each of these methodologies was applied using the software package Weka 3.6.9 [55], within which appropriate parameters were selected for SVM, C4.5 and RF using GridSearch with 10-fold cross validation settings. Logistic regression was selected for its popularity in classification models using non-censored data and in clinical settings [18, 56].

Support vector machines, originally presented in [57], find separating boundaries between decision classes after input vectors are non-linearly mapped into a high dimensional feature space. Support vector machines have been investigated in survival analysis applications [58] as they—similar to the RSA-based methods—automatically incorporate non-linearities and do not make *a priori* assumptions about factor interactions. SVM-based models are known to perform well at classification tasks, however they do not provide clinician-interpretable justification for their results [59]. Support vector machines were selected to evaluate whether the increased accessibility of the RSA-based methods involves a trade-off in accuracy.

C4.5 is a well known algorithm for generating a decision tree using information entropy to select the best splitting criteria at each node [60]. A decision tree built by C4.5 can be expressed as a set of if-then decision rules, thus providing a comparative decision rule based method. Decision trees were obtained using the Weka J48 implementation [60] of the C4.5 algorithm.

Random forests is a popular ensemble classification method based on decision trees [61]. The random forests algorithm builds an ensemble of decision trees, where each tree is built on bootstrap samples of training data with a randomly selected subset of factors.

#### Performance evaluation methods

The performance of the models was tested by measuring the discriminatory power of both the *m*- and *l*-consistent decision rules sets when applied to the reserved testing data. For our notation, a classification of *d*.*6months*=*Yes* is referred to as a positive classification, and *d*.*6months*=*No* is negative. Sensitivity is defined as the fraction of patients who did not survive six months and are correctly classified by the model, or the fraction of true positive classifications of all test patients who did not survive six months. Conversely, specificity is defined as the fraction of patients who did survive six months and were correctly classified by the model, or the fraction of true negatives of all test patients who did survive six months.

The overall accuracy of the classification models is reported in terms of area under the receiver operating characteristic (ROC) curve, or AUC (area under the curve). The ROC curve graphs the sensitivity of the classifier, or the true positive rate, versus 1 − specificity, the false positive rate, as the threshold probability, *τ*, for positive classification is varied from 0 to 1. The best overall classification performance is realized when AUC is equal to 1, while an AUC of 0.5 indicates a classifier performance no better than random selection. Best separation between decision classes is realized at the threshold corresponding to the point on the ROC curve closest to the point (0,1).

In order to select the most appropriate MODLEM and VC-DomLEM-based models for comparison, two performance issues related to the generated rule set were considered: coverage and AUC of the model. The coverage of the classification model is defined as the percentage of testing set patients for whom a classification is possible. Additionally, to evaluate the number of rules that would fire for an unseen patient, we collected information on the number of rules matching each test case patient for the evaluated levels of *m* and *l*.

Cohen’s Kappa coefficient was computed for both the selected RSA-based models and the comparative models [62]. Cohen’s Kappa coefficient is designed to measure the agreement between two classification methods, but it is commonly used to measure model performance by comparing a classifier with a random allocation of patients among the decision classes. A value of zero indicates classification accuracy equivalent to chance (zero disagreement).

Performance of the prognostic models was evaluated using a 5-fold cross validation procedure [63] wherein training and testing sets are repeatedly selected. Cross validation is a well known method that provides a reasonable estimate of the generalization error of a prediction model. In 5-fold cross validation, the entire dataset is randomly divided into five subsets, or folds, and then each fold (20 % of the dataset) is used once as a testing set, with the remaining folds (80 %) used for training.

## Results

This section presents the results obtained using MODLEM, VC-DomLEM, logistic regression, SVM, C4.5 and RF models for six-month life expectancy prognostication of terminally ill patients. The results are analyzed and compared.

**Fig. 3:**
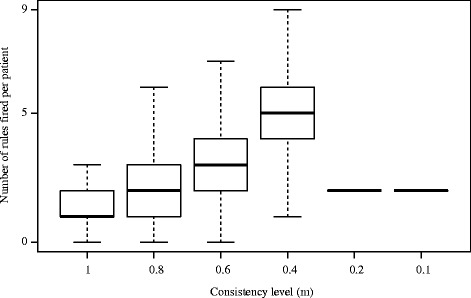
Number of rules fired in each test case for *m*-consistent MODLEM classifiers

**Fig. 4:**
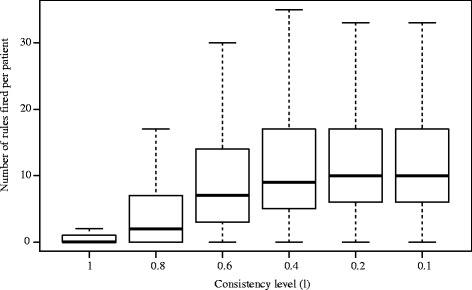
Number of rules fired in each test case for *l*-consistent VC-DomLEM classifiers

In order to select appropriate *m* and *l* values for MODLEM and VC-DomLEM-based models, respectively, the performance of these models was evaluated first. AUC and coverage for each evaluated *m* and *l* level are shown in Table 4. Figures 3 and 4 display the number of rules that fire for each patient in the five testing folds for each *m* and *l* value. Based on these results, *m*=*l*=0.6 was chosen as the rule consistency parameter for both algorithms for further evaluation with the comparative methods.

**Table 4:** AUC and coverage for MODLEM and VC-DomLEM algorithms with *l* and *m*-consistent rules

	MODLEM		VC-DomLEM	
*m,l*	AUC	Coverage (%)	AUC	Coverage (%)
0.1	0.6646	100.00	0.7280	99.88
0.2	0.6646	100.00	0.7279	99.87
0.4	0.6888	100.00	0.7277	99.65
0.6	0.6974	97.41	0.7173	98.72
0.8	0.6419	86.72	0.7093	76.85
1.0	0.6158	80.08	0.6559	35.89

The quality of approximation is 0.9244 for the CRSA, 0.3110 for the DRSA and finally 0.9014 for the VC-DRSA where the object consistency parameter *l*=0.6.

Table 5 describes the number of rules and the number of descriptors in each rule for the two rough set approach-based classifiers at the selected consistency level of 0.6. The average number of MODLEM decision rules in the five rule sets generated by cross validation is 773 rules, with mean and maximum length of 3.65 and 8 descriptors, respectively. In Fig. 3, it can be seen that at rule consistency levels of *m*=0.2 and *m*=0.1, the number of rules fired per patient is always 2. This is because the rule set is generated by only two attributes and each rule contains only one attribute in the antecedent. The VC-DomLEM decision rules are on average slightly longer, with mean and maximum length of 6.85 and 13 elementary conditions, respectively. The mean total number of VC-DomLEM rules is 1,095 rules.

**Table 5:** Number of descriptors and rules in MODLEM and VC-DomLEM induced decision rule sets, for *m*=*l*=0.6 consistent rules, across the five cross validation folds

		Descriptors in rules		
Method	Mean number of rules	Min.	Max.	Mean
MODLEM	773	1	8	3.65
VC-DomLEM	1095	2	13	6.85

For SVM, the gamma (*γ*) and cost parameter (*C*) were evaluated between 10^−1^ and 10^5^ at increments of 10^−1^; final selected parameters were *γ*=0.1 and *C*=100. For RF, the number of trees was explored between 10 and 1,000 trees at intervals of 10; the optimal number of trees thus obtained was 500. The maximum number of attributes selected at each bootstrap iteration was also explored in the range of 1 to 15 attributes, with best performance observed when the number of attributes was limited to 1. In the case of C4.5, the confidence factor used for pruning was evaluated between 0.1 and 0.9 with increments of 0.1 and 0.5 was selected. The minimum number of instances per leaf for the C4.5 decision tree was explored in steps of 1 between 1 and 100, with best performance achieved with a minimum of 40 instances per leaf. The pruned C4.5 trees contained an average of 74 nodes over the 5 cross validation folds.

The performance of all of the evaluated classification models is shown in Table 6, where Cohen’s kappa coefficient [62] and AUC are reported for each classifier, averaged over the 5 cross validation folds. Highest average kappa coefficient was achieved by RF with $\bar {\kappa } = 0.37$. Second highest average kappa coefficient was achieved by VC-DomLEM, logistic regression and SVM at $\bar {\kappa } = 0.35$. The MODLEMand C4.5 classifiers achieved $\bar {\kappa } = 0.32$ and 0.31, respectively. Average sensitivity and specificity for each of the models are also shown in Table 6. For each model and cross validation fold configuration, the sensitivity and specificity were recorded at the threshold at which both values are simultaneously maximized. This threshold is equivalent to the point on the ROC plot closest to the upper left corner and represents the point of maximum accuracy of the model.

**Table 6:** Summary of performance evaluation results of the classification models, averaged over the 5 cross validation folds, with standard deviations

Method	AUC	Kappa	Sensitivity	Specificity	Threshold (*τ*)
VC-DomLEM	0.7173 (0.014)	0.35 (0.03)	0.6391 (0.042)	0.7175 (0.033)	0.4234 (0.045)
MODLEM	0.6974 (0.015)	0.32 (0.03)	0.6447 (0.038)	0.6862 (0.037)	0.4597 (0.042)
C4.5	0.7088 (0.018)	0.31 (0.04)	0.6078 (0.055)	0.7254 (0.070)	0.4531 (0.095)
RF	0.7459 (0.014)	0.37 (0.02)	0.6384 (0.044)	0.7388 (0.039)	0.4872 (0.022)
Log. Reg.	0.7421 (0.009)	0.35 (0.01)	0.6374 (0.055)	0.7282 (0.058)	0.4715 (0.050)
SVM	0.7352 (0.009)	0.35 (0.02)	0.6526 (0.050)	0.7132 (0.040)	0.4056 (0.034)

## Discussion

All of the methodologies show fair classification accuracy given that Kappa coefficients are in the range of 0.20 to 0.40 [64]. The results presented in Table 6 show that all of the methods have similar AUC with the best performing algorithm being RF (AUC = 0.7459) and the worst being MODLEM (AUC = 0.6974). The best performing method among the decision- and rule-based methods was VC-DomLEM with an average AUC of 0.7173.

With respect to MODLEM and VC-DomLEM, *m* and *l* are clearly critical values in determining model performance. Together, Table 4 and Figs. 3 and 4 demonstrate that selecting *m*=*l*=0.6 balances the accuracy and coverage achieved by the rough set based classifiers against the amount of inconsistency allowed in each. In the case of MODLEM, *m*=0.6 is associated with highest AUC and acceptable coverage. However, in the case of VC-DomLEM, reducing *l* below 0.6 provides only marginal benefits in terms of AUC and coverage but greatly increases the amount of inconsistency allowed in the generated rules.

The quality of approximation for the CRSA classifier is 0.9244. The difference between the quality of approximation in the CRSA and the DRSA is the inclusion of the preference-ordering information, determined by the APACHE III scores. In the case of the DRSA, a strict application of this information in determining the lower approximation leads to few patients in the lower approximations, thus reducing the overall quality of approximation. Consequently, decision rules generated from this approximation are too specific and less suitable for generalizing to the classification of new cases. It is therefore reasonable to relax the conditions for assignment of objects to lower approximations. Thus, using the VC-DRSA and setting the object consistency parameter *l*=0.6, results in an improved quality of approximation of 0.9014.

All of the rule- or decision-tree-based methods demonstrated somewhat reduced performance when compared with the non-rule based classifiers. The worst-performing rule-based method, MODLEM, had an AUC 0.049 below the best-performing method, RF (0.6974, MODLEM, vs. 0.7459, RF). In contrast, VC-DomLEM demonstrated average AUC much closer to that of RF, with an average difference of only 0.029 (0.7173, VC-DomLem, vs. 0.7459, RF). In practice, this relatively small difference in performance is likely to be outweighed by the accessibility of the rule-based format of VC-DomLEM, while such benefits would be less justified in the case of MODLEM.

### Interpretation and usability of decision rules

Clinical credibility in prognostic models depends in part on the ease with which physicians and patients can understand and interpret the results of the models, in addition to the accuracy of the information they provide. The RSA-based prognostic models present the physician with a list of matched decision rules, offering significant advantages by increasing both the traceability of the model and the amount of information included in its results. This advantage is further increased in the case of VC-DomLEM, where dominance-based decision rules permit greater information density per rule by including attribute value ranges in each rule.

Table 7 contains the decision rules that fire for an example patient selected from the SUPPORT data set. This patient was 41 years old with a primary diagnosis of coma. The patient displayed moderate head injury on the Glasgow Coma Scale, elevated levels of creatinine (1.60 mg/dL) and respiratory rate (26 bpm), normal levels of sodium (133 mEq/L), low white blood cell count (1.90 cells/nL) and mean blood pressure of 107 bpm. Both the MODLEM and VC-DomLEM classifiers correctly predict that the patient will not survive six months (the patient in fact survived only 4 days).

**Table 7:** Selected decision rules from the CRSA using MODLEM and the VC-DRSA using VC-DomLEM

		RHS	
CRSA rules using MODLEM	LHS	*d.6months* =*No*	*d.6months* =*Yes*
1. If *age_score*^a^ = 0	969	593 (61 %)	376 (39 %)
2. If *scoma* = Moderate	1016	399 (39 %)	617 (61 %)
3. If *dzgroup* = Coma	465	119 (26 %)	346 (74 %)
4. If *hrt_score*^b^ = 0 AND	47	11 (23 %)	36 (77 %)
*resp_score*^c^ = 6 AND *wbc_score*^d^ = 5			
VC-DRSA rules using VC-DomLEM			
5. If *dzgroup* = Coma AND	51	4 (8 %)	47 (92 %)
*crea_score*^e^ ≥4 AND *sod_score*^f^ ≥2			
6. If *dzgroup* = Coma AND	8	8 (100 %)	0 (0 %)
*scoma* ≤ Moderate AND			
*hday* ≤ Short AND *age_ score*^a^ ≤0			

^a^*age_score:* 0=(*age*≤44)

^b^*hrt_score:* 0=(50≤*hrt*≤99)

^c^*resp_score:* 6=(25≤*resp*≤34)

^d^*wbc_score:* 5=((1≤*wbc*≤2.9) or (*wbc*≥25))

^e^*crea_score:* ≥4=(*crea*≥1.5)

^f^*sod_score:* ≥2=((*sod*≤134) or (*sod*≥155))

The VC-DomLEM classifier predicts *d.6months = Yes* with an associated score of 80 %, based on the two rules (Rules 5 and 6). As can be seen in Table 7, Rule 5 isolates the combination of Coma and elevated creatinine and sodium levels as a key predictor of six-month survival. In the case of Rule 5, 51 patients in the training set have similar conditions as the example patient, of which 47 did not survive six months. On the other hand, Rule 6 somewhat counterbalances this prediction, pointing to 8 young patients with moderate coma who have been in the hospital less than 44 days, of whom all 8 survived six months.

The MODLEM classifier provides a less specific prediction, classifying the example patient as not surviving six months with an associated score of 55 %. Upon further investigation, the rules matching the example patient (Rules 1–4) are more general than the rules provided by the VC-DomLEM classifier. Rules 1–3 provide general rules that point to the age, level of head trauma and primary diagnosis of the patient. Considering only these three rules, the associated score would be *d.6months = Yes* with a score of 54 %, but this score is revised slightly by Rule 4 further in favor of *d.6months = Yes*. Rule 4 isolates normal average heart beat, high respiratory rate and low (and also very high) white blood cell counts.

For all of the classifiers, a final prediction and associated score are presented by the classifier. However, only in the case of MODLEM and VC-DomLEM is the prediction further supported by the set of rules from which said prediction derived. Thus, the gestalt survival expectation is presented without loss of contradictory information, providing the physician with both the prognostication as well as supporting and contradicting information. In contrast, while a decision tree obtained using C4.5 can be represented as a set of rules, only a single rule representing the matching terminal node is returned to the physician. Among the rule-based methods, those rules derived from the VC-DRSA naturally include attribute value ranges for which the rule is valid, succinctly providing even more information to the physician and further increasing the utility of the life expectancy prediction. In a clinical setting, this set of rules serves to support clinical decisions for future treatment or palliative care strategies as well as to support the explanation of these decisions to the involved patient and their family.

Decision tree models offer the additional benefit of visually representing the entire model in a single structure, and given their hierarchical structure can be used to guide the decision process of the physician [65]. Decision trees models are most useful when built with the input of domain experts as pruning visually complex decision trees must balance tradeoffs between accuracy and simplicity [66]. Many methods exist for the visualization of decision trees and the performance of visually-tuned decision trees may be comparable to more complex versions of the same model [67].

A further benefit of the rule-based methods is that the rules clearly indicate the patient characteristics most relevant to their survival expectation. This increases the transparency and interpretability of the classification process, strengthening the accessibility, and hence credibility, of the model. Additionally, the decision rules do not individually involve all of the condition attributes. This offers the advantage of providing potentially acceptable results should a particular prognostic factor be difficult or too costly to ascertain for a patient [34].

This is in stark contrast to SVM, neural networks, and other black-box methods where very little insight is available to a decision maker as to how an outcome was predicted. While similar performance in terms of accuracy was seen for all of the classification models, the rule-based models naturally express results in terms of a set of decision rules, a benefit that is not present in logistic regression, RF, or the mentioned black-box methods. As an ensemble method, the RF method functionally reduces to a black-box style model, despite its use of decision trees.

### Decision analysis for hospice referral

Consider the costs—economic, emotional and physical—associated with the decision to enter hospice care. These costs are justified for patients who either enter hospice care at the appropriate time or for those who do not enter hospice care when they could benefit from curative treatment. These cases represent true positive and true negative classifications. A higher emotional and physical cost is born by patients sent to hospice care but who ultimately survive six months—a false positive. The highest cost of all, emotionally, economically and physically is born by the patient and his or her family when costly treatment is prolonged for a patient who should have been referred to a hospice care program—a false negative. In this last case, some or all of the benefits of hospice care would be lost while the stresses and economic burden of aggressive treatment are endured.

In this light, the threshold parameter, *τ* (described in section Decision rules), can be seen as a representation of the patient and family’s preference for hospice care treatment and their risk tolerance for a mistaken referral. The threshold parameter relates sensitivity to specificity and stipulates the required level of certainty for a positive classification. A higher threshold value requires a higher probability of not surviving six months for the classification of a patient as a hospice candidate, decreasing the sensitivity and increasing specificity (indicating a preference for continued treatment). Conversely, a lower threshold value increases sensitivity while reducing specificity, indicating a preference for avoiding the costly mistake of unnecessary treatment.

As this threshold value is a subjective matter and varies between physicians, patients and family members, one suggested approach [68] involves the measurement of the amount of regret the decision maker would have should an incorrect decision be made. As medical decisions must take into account the preferences of those ultimately affected by the decision, this application of regret theory allows for the formal treatment of those preferences by calculating the threshold value as a function of the measured anticipated regret.

## Conclusions

This paper contributes to the growing body of research in RST—and its extensions—as a prognostic modeling framework and highlights the strengths of this approach in terms of accessibility. The non-rule-based methods—RF, logistic regression, and SVM—were found to more accurately predict death within six months, however the benefits of the rule-based methods may outweigh the performance differential, particularly in the case of VC-DomLEM where this difference was small. The intuitive structure of the rough set approaches, built on indiscernibility and dominance relations and expressed in terms of if-then decision rules, offers both more insight into the modeling process and more opportunity for the knowledge extraction process to incorporate the personal preferences of those making and being affected by the decision.

The performance of the classifiers presented in this study is good but sub-optimal, indicative of a challenging problem in need of further research. The increased performance achieved by the variable consistency approach suggests a dataset of highly diverse patients. Future research will explore methods to improve the overall classifier performance and address this diversity by building localized models for patient subgroups using rough sets concepts to group patients with similar differentiating characteristics.

A recent study developed a six-month survival prognostic model primarily based on the Medicare Health Outcomes Survey responses of community-dwelling elderly patients [69]. This model, named the Patient-Reported Outcome Mortality Prediction Tool (PROMPT), achieved comparable AUC using only basic medical information, indicating that the performance of classification models for six-month survival is still a major issue for the targeted domain of hospice referral recommendation.

An important limitation of this study is that patient-specific disease progression over time is not considered, in part due to the static nature of the data set used. Future research must address the temporal aspect of disease progression, a consideration often missing in other prognostic models for hospice referral. The progression of a terminal illness is often highly non-linear by nature and generally does not present as a steady decline over time but rather as periods of relative stability marked by turning points of acute decline. A prognostic model that takes into account this temporal aspect may possibly provide both more accurate life expectancy prognoses and more useful information for palliative care planning.

## Availability of supporting data

The data set supporting the results of this article is publicly available at http://biostat.mc.vanderbilt.edu/wiki/Main/SupportDesc.
